# Effect of tannins and cellulase on growth performance, nutrients digestibility, blood profiles, intestinal morphology and carcass characteristics in Hu sheep

**DOI:** 10.5713/ajas.18.0901

**Published:** 2019-03-07

**Authors:** M. D. Zhao, L. F. Di, Z. Y. Tang, W. Jiang, C. Y. Li

**Affiliations:** 1 Department of Animal Science, Agricultural College of Yanbian University, Jilin 133002, China; 2 Innovation Center of Beef Cattle Science and Industry Technology, Yanbian University, Jilin 133002, China

**Keywords:** Tannins, Cellulase, Growth Performance, Nutrient Digestibility, Blood Profiles, Intestinal Morphology

## Abstract

**Objective:**

This study was conducted to evaluate the effects of tannins and cellulase on growth performance, nutrient digestibility, blood profiles, intestinal morphology, and carcass characteristics in Hu sheep.

**Methods:**

A total of 48 three-month-old meat Hu sheep (25.05±0.9 kg) were blocked based on body weight, and randomly allotted to 4 treatments with 3 replicates of 4 sheep each. The experiment lasted for 80 d, and dietary treatments were as follows: i) CON, control diet; ii) TAN, CON+0.1% tannins; iii) CEL, CON+0.1% cellulase; iv) TAN+CEL, CON+0.1% tannins and 0.1% cellulase.

**Results:**

Compared with CON, CEL, and TAN+CEL had greater (p<0.05) final body weight (FBW) and average daily gain but lower (p<0.05) feed conversion ratio, while FBW of TAN+ CEL was lower (p<0.05) than that of CEL. The apparent total tract digestibility (ATTD) of dry matter in TAN, CEL, and TAN+CEL groups were higher (p<0.05) than that in CON. CEL and TAN+CEL groups had greater (p<0.05) ATTD of crude fiber compared with TAN and CON, while TAN group had lower (p<0.05) ATTD of crude protein than other treatments. TAN, CEL, and TAN+CEL groups increased (p<0.05) serum globulin and alkaline phosphatase but decreased (p<0.05) albumin/globulin. Serum total protein was greatest for TAN+CEL, intermediate for TAN and CEL and least for CON (p<0.05). TAN+CEL group increased (p<0.05) dressing percentage compared with CON, while the backfat thickness of CEL was lower (p<0.05) than that of CON. The villus height of jejunum and ileum in CEL and TAN+CEL groups were greater (p<0.05) than that in CON, and the crypt depth and villus height: crypt depth of jejunum were increased (p<0.05) in TAN, CEL, and TAN+CEL groups.

**Conclusion:**

The addition of tannins and cellulase together promoted nutrient digestion, liver protein synthesis and intestinal development and thus improved growth performance and carcass characteristics.

## INTRODUCTION

Tannins are a group of polyphenolic compounds generally occurring in the forages consumed by ruminants [1]. Researches have shown that tannins improved protein utilization in ruminants because they could complex with the proteins and protect them from degradation, and then release the protein later in the intestine [2]. Therefore, the amount of high-quality protein available to ruminants for absorption would be increased [3]. Moreover, feeding tannin-containing forages or tannins supplementation reduced CH_4_ and N_2_O emissions and increased the amount of energy available to the ruminants. It is reported that tannins could reduce total methanogen populations and methane emissions [4].

In ruminants, fiber digestion was a limiting factor in nutrient utilization and ruminant production systems [5]. Exogenous cellulase supplementation has been proved to be an effective mean of improving nutrient utilization of forages and yielding more metabolizable energy for ruminants [5]. Previous studies demonstrated that although the results were inconsistent, the addition of cellulase could increase nutrient digestibility and feed conversion [6].

However, tannins could interact with the cell wall and secrete catabolic enzymes and then inhibit cellulolytic microorganisms in the rumen, and ultimately reduce the secretion of cellulase [7]. Moreover, previous study proved that tannins presented enzyme-specific interactions with cellulase to inhibit its activity. As a result, fiber digestion would be interfered by tannins [8].

An *in vitro* experiment showed that cellulase addition decreased soluble tannins and condensed tannins with increasing enzyme inclusions [9], which might counteract the reduction of cellulase. However, reports about the effects of tannins or cellulase alone or together on sheep were very limited. Thus, we hypothesized that the addition of tannins and cellulase together might have a beneficial effect in sheep. Therefore, the objective of this study was to determine the effects of tannins and cellulase on growth performance, nutrients digestibility, blood profiles, intestinal morphology and carcass characteristics in Hu sheep.

## MATERIALS AND METHODS

### Animals, diets, and treatments

All animal experiment procedures in this study were approved by the Institutional Animal Care and Use Committee of Yanbian University. The experiment was conducted at Animal Husbandry Research Institute of Yanbian Academy of Agricultural Sciences from May 2018 to September 2018. A total of 48 three-month-old meat Hu sheep (25.05±0.9 kg) were blocked based on body weight and randomly allocated into 4 treatments with 3 pens per treatment and 4 sheep per pen. Prior to the experiment, the animals were treated against internal and external parasites. Experimental treatments included: i) CON, control diet; ii) TAN, CON+0.1% tannins (95% purity); iii) CEL, CON+0.1% cellulase (15,000 IU/g); iv) TAN+ CEL, CON+0.1% tannins and 0.1% cellulase. After an adaptation period of 14 days to the experimental conditions, the sheep stayed for 80-d feeding trial. Diets were formulated to provide all the nutrients to meet or exceed NRC requirements [10]. All diets were kept at a concentrate: roughage ratio of 50:50, where the roughage component was maize straws and the concentrate consisted of corn, wheat bran, soybean meal and premix (Table 1). The diets were offered to the animals in two equal halves at 7:30 and 15:30 daily. Animals were allowed *ad libitum* access to feed and water throughout the experiment.

### Growth performance

The sheep were weighed individually on d 1 and 80 of the experiment. Average daily gain (ADG) was calculated by the difference between final body weight (FBW) and initial body weight. Feed offered and refused was quantified daily for each pen to calculate average daily feed intake (ADFI). Feed conversion ratio (F/G) was calculated as a ratio of ADFI to ADG.

### Nutrient digestibility

Digestibility trial was conducted at the end of the feeding trial and feces were collected daily for 6 days from a fecal collection bag attached to the sheep. The feces were weighed and mixed, and 25% of representative samples were taken and frozen at −20°C. At the end of the collection period, all samples of each sheep were pooled and 25% of the composite sample was taken and dried at 65°C for 72 h. Daily feed per animal was also collected. The chemical analysis of feces and feed were performed to determined dry matter (DM), crude protein (CP), ether extract (EE), crude fiber (CF) and ash according to standard AOAC method [11]. Apparent total tract digestibility (ATTD) of DM and other nutrients was determined as a percentage of the nutrient intake not recovered in the feces.

### Blood profiles

At the end of feeding trial, blood samples (10 mL) via the jugular vein were collected from three sheep from each treatment. The blood samples were stored at −4°C and then submitted for biochemical parameters analysis. The albumin (ALB), globulin (GLB), albumin/globulin (A/G), total protein (TP), total cholesterol (TC), triglyceride (TG), alanine transaminase (ALT), alkaline phosphatase (ALP), glucose (GLU) concentrations were determined by an automatic biochemistry analyzer (Hitachi Model 7600, Tokyo, Japan).

### Carcass characteristics

After blood collection, the same sheep were weighted and euthanized with an intravenous injection of 200 mg/kg of body weight (BW) sodium pentobarbital (Sigma Chemical Co., St. Louis, MO, USA) after 24 h fasting. Decapitation and skinning of the sheep were undertaken after removing legs below the hock and knee joints. The carcass weight was checked after removing thoracic, abdominal and pelvic contents, and the organs (heart, liver, lungs, and kidneys) were weighed. Dressing percentage was calculated as carcass weight divided by the live body weight. The carcass was split between 12th and 13th ribs perpendicular to the backbone to measure the cross section of rib eye muscle area and the backfat thickness over the maximum muscle depth at same site was also recorded.

### Tissue collection and measurement

After abdominal contents collection, 2 to 2.5 cm longitudinal sections of jejunum and ileum were taken from 5 cm distal to the duodenojejunal junction and jejunoileal junction separately. All samples were soaked in 4% paraformaldehyde fix solution immediately and then embedded in paraffin and sectioned (3 to 5 μm). Slides were stained with hematoxylin and eosin (H&E) as described in Holle et al [12], then digitized and measured by a fluorescence microscope camera via DP2-BSW software (Olympus, Tokyo, Japan). The crypt depth (CD), villus height (VH), and VH:CD ratio was measured according to methods described by McLeod et al [13]. For each section, 12 of the most representative villi and crypt were used for measurement.

### Statistical analysis

Data were analyzed using the general linear model Procedure of SAS as a randomized complete block design (SAS Inst. Inc., Cary, NC, USA) with the pen being considered as the experimental unit. The average initial BW was used as a covariate for ADFI and ADG. Differences among treatment means were determined using Tukey’s range test. Variability in the data was expressed as the standard error means and a probability level of p<0.05 was considered statistically significant.

## RESULTS

### Growth performance

CEL and TAN+CEL groups increased (p<0.05) FBW compared with CON, and the FBW of CEL group was higher (p< 0.05) than that of TAN+CEL group (Table 2). ADG did not differ (p>0.05) between CEL and TAN+CEL group but both were higher than CON and TAN group (p<0.05). There were no significant differences in ADFI among treatments (p>0.05). F/G of CEL group was lower (p<0.05) than that of CON group. TAN and TAN+CEL groups had no significant effects on F/G compared with CON.

### Nutrient digestibility

ATTD of DM in TAN, CEL and TAN+CEL groups were greater (p<0.05) than that in CON (Table 3). CEL and TAN+CEL groups had higher (p<0.05) ATTD of CF compared with TAN and CON, while TAN group had lower (p<0.05) ATTD of CP than other treatments. ATTD of EE or ash did not differ (p>0.05) among groups.

### Blood profiles

Compared with CON, TAN, CEL, and TAN+CEL groups increased (p<0.05) serum GLB and ALP but decreased (p< 0.05) A/G (Table 4). TP was greatest for TAN+CEL group, intermediate for TAN and CEL group, and least for CON (p<0.05). No differences were observed (p>0.05) in ALB, TC, TG, ALT, or GLU among treatments.

### Intestinal morphology

The VH of jejunum and ileum in CEL and TAN+CEL groups was greater (p<0.05) than that in CON, and no differences were observed (p>0.05) between TAN and CON (Table 5). Compared with CON, the CP and VH:CD of jejunum were increased (p<0.05) in TAN, CEL, and TAN+CEL groups. However, no significant differences in the CD and VH:CD were found (p>0.05) for the ileum.

### Carcass characteristics

The slaughter body weight of CEL group was higher (p<0.05) than that of CON and no differences were observed (p>0.05) among the other three groups (Table 6). The carcass weight did not (p>0.05) differ between CEL and TAN+CEL group but both were greater than CON (p<0.05). TAN+CEL group significantly increased (p<0.05) dressing percentage compared with CON but did not (p>0.05) differ from TAN or CEL groups. The backfat thickness of TAN was lower (p<0.05) than that of CON. No significant differences were found (p> 0.05) for eye muscle area or weight of heart, liver, lungs, and kidneys.

## DISCUSSION

It has been generally accepted that tannins might cause a reduction of daily feed intake and weight gain [14]. However, FBW, ADG, ADFI, and F/G were not affected by tannins supplementation alone in current study. A meta-analysis study indicated that tannins did not have negative effect on DM intake or live weight change in growing sheep, which was consistent with results in the present study [15]. Compared with CON, the cellulase supplementation alone increased FBW and ADG, but reduced F/G in the current study. Similarly, calves fed cellulase had higher FBW, ADG and feed efficiency [5]. The mixture of tannins and cellulase increased FBW and ADG compared with CON in the present study. However, the sheep fed tannins and cellulase together had lower FBW than those fed cellulase alone. The results suggested that the cellulase supplementation alone improved growth performance but tannins may partly interfer.

As is well known, tannins have an ability to complex with protein to protect it against ruminal degradation, and the protection was more effective for proteins than DM or other nutrients [16]. In the present study, tannins supplementation alone increased DM digestibility and decreased CP digestibility. Similarly, it is noted that quebracho tannin extract in diets decreased apparent digestibility of CP in cattle [17]. However, pervious researches also showed that tannins supplementation would decrease DM digestibility in ruminants [15]. These inconsistent results might be attributed to the different origins of tannins, which vary greatly in their capacity to bind carbohydrates [18]. The increased digestibility of DM and CF noted with cellulase supplementation alone in the current study agreed with the results of other studies. It is found that lambs fed exogenous cellulase diets had greater DM and CF digestibility than those fed non-cellulase diets [5]. *In vitro* research also proved that exogenous cellulase supplementation alone increased substrate fiber degradation, volatile fatty acid production, and ruminal microbial growth to enhance rumen fermentation [19]. This improvement could ultimately increase DM and CF digestibility and might partly explain the increase of ADG and reduction of F/G. The mixture of tannins and cellulase supplementation increased DM and CF digestibility compared with CON and had greater CP digestibility than tannins group. As mentioned above, the results indicated that tannins in the present study might not inactivate the cellulase, and the effect of cellulase could partially counteract the reduction of CP digestibility by tannins.

Serum TP was a marker of nutritional level and was increased by tannins and cellulase in present study [20]. The results revealed that liver protein synthesis and rumen microbial protein production might be improved by tannins and cellulase, even when the CP digestibility was decreased by tannins. Similarly, previous study found that supplementing lambs’ diets with fiberolytic enzymes significantly increased serum TP compared with CON [21]. Furthermore, the mixture of tannins and cellulase had greater serum TP than other treatments. As mentioned above, tannins and cellulase together increased CP digestibility compared with tannins alone and the cellulase could eliminate the side effect of tannins on the CP digestibility, which might contribute to the higher serum TP. Therefore, the results suggested that the improvement of serum TP may be enhanced by the interaction of tannins and cellulase. Moreover, the increased serum GLB contributed to the increase of serum TP and decrease of A/G in the current study. Serum GLB played an important role in immune system and the increased GLB could enhance humoral immune response against pathogenic viruses and microorganisms [22]. Therefore, the increased serum GLB indicated that tannins, cellulase and the mixture could improve immunity of sheep. Besides, the ALP was increased by tannins, cellulase and the mixture, which played an integral role in metabolism within the liver and development within the skeleton [23]. The results revealed that liver function and skeleton development might be improved by tannins and cellulase. However, the GLB and ALP were not increased by the mixture compared with tannins or cellulase alone. The results indicated that there was not a synergy between tannins and cellulase on improving serum GLB and ALP.

In the present study, the mixture of tannins and cellulase increased carcass weight and dressing percentage compared with CON, which indicated that the addition of tannins and cellulase together could improve slaughter carcass characteristics. On the contrary, feeding xylanase and cellulase had no effect on hot carcass weight and dressing percentage of steers [24]. Tannins in birdsfoot trefoil did not affect carcass weight or dressing-out of lambs [25]. The differences might be due to different species and tannins origins. Besides, the backfat thickness was decreased by tannins alone. Similarly, finishing steers fed high-tannin sorghum diets had lower backfat thickness than CON [26]. The results illustrated that tannins had adverse effect on backfat and the cellulase could eliminate the side effect. The tannins and cellulase increased liver, heart and kidney weight numerically, which was consistent with previous study [21]. The results showed that the mixture of tannins and cellulase might promote organs development, which may have contributed to increased ADG and the changes in blood profiles.

The VH, CD, and VH:CD ratio are markers of bowel integrity and increase during development [27]. In the present study, the CD of jejunum was decreased and VH:CD was increased by tannins. However, feeding 0.5% quebracho tannin induced ulceration and an increase in mucosal histiocytes in the jejunum and ileum of sheep [28]. The differences might be due to the higher tannins content in their diets compared with the present study. Our results indicated that 0.1% tannins could promote jejunum development. The cellulase increased VH of jejunum and ileum and VH:CD of jejunum, but decreased VH of jejunum in the current study. The results indicated cellulase could promote jejunum and ileum development and ultimately improve nutrient digestion and absorption, which may contribute to the higher ADG and feed efficiency [27,29]. The mixture of tannins and cellulase also decreased VH of jejunum, but increased VH and VH:CD of jejunum, especially the VH of ileum. The results suggested that there was a synergy between tannins and cellulase on the development of ileum. However, no significant differences in the CD or VH:CD was found for the ileum, illustrating that the morphology of the jejunum was more easily affected by tannins and cellulase than that of the ileum during growth [27].

## CONCLUSION

The tannins supplementation alone increased ATTD of DM, serum GLB and TP, CD, and VH:CD of jejunum, but decreased ATTD of CP and backfat thickness. The cellulase supplementation alone increased ADG, feed efficiency, ATTD of DM and CF, serum TP, GLB, and ALP, VH and VH:CD of jejunum and VH of ileum, while decreased serum A/G and CD of jejunum. The mixture of tannins and cellulase improved ATTD of CP, serum TP and CD of ileum, but decreased FBW compared with tannins or cellulase alone. The results illustrated that although CP digestibility and FBW may be negatively affected by tannins, the mixture of tannins and cellulase might promote nutrient digestion, liver protein synthesis and intestinal development and thus improved growth performance and carcass characteristics.

## Figures and Tables

**Table 1:** Dietary ingredients and calculated nutritional composition of the control diet

Item	Content
Ingredient (% of DM)	
Corn	60
Wheat bran	11
Soybean meal	27
Premix1)	2
Chemical composition (% of DM)	
ME (MJ/kg)	9.48
CP	18.71
Ca	0.50
P	0.46

DM, dry matter; ME, metabolizable energy; CP, crude protein.

1) Each kilogram contains: vitamin A 100,000 IU; vitamin D_3_ 80,000 IU; vitamin E, 700 IU; iron, 3.5 g; copper, 0.9 g; manganese 3 g; zinc, 5.5 g; iodine, 60 mg; selenium, 30 mg; cobalt, 45 mg; calcium, 20%; total phosphorus, 3.5%; chloride sodium, 30%.

**Table 2:** Effects of tannins and cellulase on growth performance in Hu sheep1)

Item	CON2)	TAN2)	CEL2)	TAN+CEL2)	SEM	p-value
IBW (kg)	25.17	25.50	25.30	24.23	0.45	0.76
FBW (kg)	35.70^c^	36.40^bc^	38.37^a^	37.27^b^	0.50	0.04
ADG (g)	131^b^	136^b^	163^a^	163^a^	5.2	0.03
ADFI (g)	2,161	2,172	2,233	2,290	52	0.37
F/G	16.44^a^	15.94^ab^	13.66^b^	14.14^ab^	0.68	0.04

SEM, pooled standard error of the means; IBW, initial body weight; FBW, final body weight; ADG, average daily gain; ADFI, average daily feed intake; F/G, feed conversion ratio.

1) Each mean represents 3 replications with 4 sheep per replication.

2) CON, control diet; TAN, CON+0.1% tannins (95% purity); CEL, CON+0.1% cellulose (15,000 IU/g); TAN+ CEL, CON+0.1% tannins and 0.1% cellulase.

a–c Mean values within a row marked with different superscript letters differ significantly at p<0.05.

**Table 3:** Effects of tannins and cellulase on nutrient digestibility in Hu sheep1)

Item (%)	CON2)	TAN2)	CEL2)	TAN+CEL2)	SEM	p-value
DM	64.47^b^	65.67^a^	66.57^a^	66.27^a^	0.38	0.03
CP	64.34^a^	61.33^b^	64.50^a^	64.03^a^	0.36	0.04
EE	53.63	54.20	54.37	54.43	0.26	0.56
CF	60.97^b^	60.37^b^	64.07^a^	63.50^a^	0.40	0.03
ASH	14.27	14.47	14.47	14.57	0.28	0.61

SEM, pooled standard error of the means; DM, dry matter; CP, crude protein; EE, ether extract; CF, crude fiber; ASH, ash.

1) Each mean represents 3 replications with 4 sheep per replication.

2) CON, control diet; TAN, CON+0.1% tannins (95% purity); CEL, CON+0.1% cellulose (15,000 IU/g); TAN+CEL, CON+0.1% tannins and 0.1% cellulase.

a,b Mean values within a row marked with different superscript letters differ significantly at p<0.05.

**Table 4:** Effects of tannins and cellulase on blood profiles in Hu sheep1)

Item	CON2)	TAN2)	CEL2)	TAN+CEL2)	SEM	p-value
ALB (g/L)	30.46	29.89	29.37	29.45	0.84	0.38
GLB (g/L)	28.58^b^	32.19^a^	34.85^a^	32.44^a^	0.69	0.02
A/G	1.07^a^	0.93^b^	0.84^b^	0.91^b^	0.03	0.03
TP (g/L)	61.47^c^	64.00^b^	63.38^b^	67.78^a^	0.81	0.02
TC (mmol/L)	1.57	1.52	1.57	1.55	0.04	0.77
TG (mmol/L)	0.42	0.41	0.45	0.37	0.05	0.50
ALT (U/L)	21.51	23.56	24.94	23.62	3.04	0.42
ALP (U/L)	250.5^b^	280.9^a^	286.2^a^	294.9^a^	8.24	0.03
GLU (mmol/L)	3.47	3.51	3.51	3.47	0.04	0.62

SEM, pooled standard error of the means; ALB, albumin; GLB, globulin; A/G, albumin/globulin; TP, total protein; TC, total cholesterol; TG, triglyceride; ALT, alanine transaminase; ALP, alkaline phosphatase; GLU, glucose.

1) Each mean represents 3 replications with 1 sheep per replication.

2) CON, control diet; TAN, CON+0.1% tannins (95% purity); CEL, CON+0.1% cellulose (15,000 IU/g); TAN+CEL, CON+0.1% tannins and 0.1% cellulase.

a,b Mean values within a row marked with different superscript letters differ significantly at p<0.05.

**Table 5:** Effects of tannins and cellulase on intestinal morphology in Hu sheep1)

Item	CON2)	TAN2)	CEL2)	TAN+CEL2)	SEM	p-value
Villus height (μm)						
Jejunum	522.8^c^	530.7^ab^	544.3^a^	540.8^bc^	5.43	0.04
Ileum	492.3^c^	505.0^ab^	508.6^b^	525.0^a^	4.25	0.03
Crypt depth (μm)						
Jejunum	240.0^a^	217.7^b^	220.8^b^	223.8^b^	4.87	0.03
Ileum	214.9	213.2	207.6	210.8	5.26	0.39
Villus height/crypt depth						
Jejunum	2.18^a^	2.44^b^	2.47^b^	2.42^b^	0.06	0.03
Ileum	2.31	2.36	2.45	2.49	0.07	0.53

SEM, pooled standard error of the means.

1) Each mean represents 3 replications with 1 sheep per replication.

2) CON, control diet; TAN, CON+0.1% tannins (95% purity); CEL, CON+0.1% cellulose (15,000 IU/g); TAN+CEL, CON+0.1% tannins and 0.1% cellulase.

a–c Mean values within a row marked with different superscript letters differ significantly at p<0.05.

**Table 6:** Effects of tannins and cellulase on carcass characteristics in Hu sheep1)

Item	CON2)	TAN2)	CEL2)	TAN+CEL2)	SEM	p-value
Slaughter body weight (kg)	35.60^b^	36.60^ab^	38.40^a^	37.40^ab^	0.98	0.03
Carcass weight (kg)	16.01^c^	17.12^bc^	19.11^ab^	19.91^a^	0.74	0.02
Dressing percentage (%)	44.97^b^	46.79^ab^	49.75^ab^	53.27^a^	2.25	0.04
Backfat thickness (cm)	1.20^a^	0.67^b^	0.80^ab^	1.03^ab^	0.19	0.03
Eye muscle area (cm^2^)	24.75	32.53	35.45	33.45	8.24	0.48
Heart weight (g)	168.8	192.9	196.8	165.7	10.53	0.29
Liver weight (g)	378.1	420.6	455.5	438.1	29.54	0.34
Lung weight (g)	398.8	367.9	360.7	372.4	18.21	0.65
Kidney weight (g)	73.05	82.95	83.30	70.70	3.76	0.51

SEM, pooled standard error of the means.

1) Each mean represents 3 replications with 1 sheep per replication.

2) CON, control diet; TAN, CON+0.1% tannins (95% purity); CEL, CON+0.1% cellulose (15,000 IU/g); TAN+CEL, CON+0.1% tannins and 0.1% cellulase.

a–c Mean values within a row marked with different superscript letters differ significantly at p<0.05.

## References

[1] Walton J, Waghorn G, Plaizier J (2001). Influence of condensed tannins on gut morphology in sheep fed *Lotus pedunculatus*. Can J Anim Sci.

[2] Bunglavan S, Dutta N (2013). Use of tannins as organic protectants of proteins in digestion of ruminants. J Livest Sci.

[3] Stewart EK, Villalba JJ, Rood KA Environmental and animal benefits when beef cattle consume condensed and hydrolysable tannins. All Current Publications 2018.

[4] Jayanegara A, Goel G, Makkar HPS, Becker K (2015). Divergence between purified hydrolysable and condensed tannin effects on methane emission, rumen fermentation and microbial population *in vitro*. Anim Feed Sci Technol.

[5] Titi HH, Tabbaa MJ (2004). Efficacy of exogenous cellulase on digestibility in lambs and growth of dairy calves. Livest Prod Sci.

[6] Bhasker TV, Nagalakshmi D, Rao DS (2013). Development of appropriate fibrolytic enzyme combination for maize stover and its effect on rumen fermentation in sheep. Asian-Australas J Anim Sci.

[7] McSweeney C, Palmer B, Bunch R, Krause DO (2001). Effect of the tropical forage calliandra on microbial protein synthesis and ecology in the rumen. J Appl Microbiol.

[8] Olsen SN, Bohlin C, Murphy L (2011). Effects of non-ionic surfactants on the interactions between cellulases and tannic acid: a model system for cellulase–poly-phenol interactions. Enzyme Microb Technol.

[9] He L, Zhou W, Wang Y, Wang C, Chen X, Zhang Q (2018). Effect of applying lactic acid bacteria and cellulase on the fermentation quality, nutritive value, tannins profile and *in vitro* digestibility of *Neolamarckia cadamba* leaves silage. J Anim Physiol Anim Nutr.

[10] National Research Council (2007). Nutrient requirements of small ruminants.

[11] AOAC (1995). Official methods of analysis of AOAC.

[12] Holle S, Birtles M (1990). An immunocytochemical method for studying patterns of cell proliferation in the wool follicle. NZ Vet J.

[13] McLeod JS, Church JT, Yerramilli P (2018). Gastrointestinal mucosal development and injury in premature lambs supported by the artificial placenta. J Pediatr Surg.

[14] Salem HB, Nefzaoui A, Makkar HPS, Hochlef H, Ben Salem I, Ben Salem L (2005). Effect of early experience and adaptation period on voluntary intake, digestion, and growth in Barbarine lambs given tannin-containing (*Acacia cyanophylla* Lindl. foliage) or tannin-free (oaten hay) diets. Anim Feed Sci Technol.

[15] Méndez-Ortiz F, Sandoval-Castro C, Ventura-Cordero J, Sarmiento-Franco LA, Torres-Acosta JFJ (2018). Condensed tannin intake and sheep performance: a meta-analysis on voluntary intake and live weight change. Anim Feed Sci Technol.

[16] Rosenthal GA, Berenbaum MR (2012). Herbivores: their interactions with secondary plant metabolites: ecological and evolutionary processes.

[17] Beauchemin KA, McGinn SM, Martinez TF, McAllister TA (2007). Use of condensed tannin extract from quebracho trees to reduce methane emissions from cattle. J Anim Sci.

[18] McAllister TA, Martinez T, Bae HD, Muir AD, Yanke LJ, Jones GA (2005). Characterization of condensed tannins purified from legume forages: chromophore production, protein precipitation, and inhibitory effects on cellulose digestion. J Chem Ecol.

[19] Giraldo LA, Tejido ML, Ranilla MJ, Carro MD (2007). Effects of exogenous cellulase supplementation on microbial growth and ruminal fermentation of a high-forage diet in Rusitec fermenters. J Anim Sci.

[20] Förhécz Z, Gombos T, Borgulya G, Pozsonyi Z, Prohászka Z, Jánoskuti L (2009). Red cell distribution width in heart failure: prediction of clinical events and relationship with markers of ineffective erythropoiesis, inflammation, renal function, and nutritional state. Am Heart J.

[21] Abutaima A (2017). Utilization of fiberolytic enzymes for improving digestibility in assaf lambs feed. PhD Thesis.

[22] Woof JM, Kerr MA (2006). The function of immunoglobulin A in immunity. J Pathol.

[23] Millán JL (2006). Alkaline phosphatases: structure, substrate specificity and functional relatedness to other members of a large superfamily of enzymes. Purinergic Signal.

[24] ZoBell DR, Wiedmeier R, Olson K, Treacher R (2000). The effect of an exogenous enzyme treatment on production and carcass characteristics of growing and finishing steers. Anim Feed Sci Technol.

[25] Douglas G, Stienezen M, Waghorn G, Foote AG, Purchas RW (1999). Effect of condensed tannins in birdsfoot trefoil (*Lotus corniculatus*) and sulla (*Hedysarum coronarium*) on body weight, carcass fat depth, and wool growth of lambs in New Zealand. NZ J Agric Res.

[26] Larraín R, Schaefer D, Arp S, Claus JR, Reed JD (2009). Finishing steers with diets based on corn, high-tannin sorghum, or a mix of both: Feedlot performance, carcass characteristics, and beef sensory attributes. J Anim Sci.

[27] Li H, Ran T, He Z, Yan Q, Tang S, Tan Z (2016). Postnatal developmental changes of the small intestinal villus height, crypt depth and hexose transporter mRNA expression in supplemental feeding and grazing goats. Small Rumin Res.

[28] Dawson JM, Buttery PJ, Jenkins D, Wood CD, Gill M (1999). Effects of dietary quebracho tannin on nutrient utilisation and tissue metabolism in sheep and rats. J Sci Food Agric.

[29] Meale SJ, Beauchemin KA, Hristov A, Chaves AV, McAllister TA (2014). Board-invited review: opportunities and challenges in using exogenous enzymes to improve ruminant production. J Anim Sci.

